# Durable response to dual immune checkpoint blockade in Lynch syndrome–associated serous ovarian carcinoma: a case report

**DOI:** 10.3389/fmed.2026.1868646

**Published:** 2026-09-01

**Authors:** Mohamad Mourad, Nada Assaf, Layal Al Mahmasani, Rami Mahfouz, Najla Fakhruddin, Ali Shamseddine

**Affiliations:** 1 Division of Hematology/Oncology, Department of Internal Medicine, American University of Beirut Medical Center, Beirut, Lebanon; 2 Department of Pathology and Laboratory Medicine, American University of Beirut Medical Center, Beirut, Lebanon

**Keywords:** deficient mismatch repair (dMMR), immune checkpoint inhibitors (ICI), Lynch syndrome, MSI-H, ovarian serous adenocarcinoma

## Abstract

**Background:**

Immune checkpoint inhibitors (ICIs) have demonstrated efficacy in microsatellite instability–high (MSI-H) or mismatch repair–deficient (dMMR) tumors. However, their role in dMMR serous ovarian cancer remains undefined, particularly when given without chemotherapy.

**Case description:**

We report the case of a 42-year-old woman with a strong family history of malignancy who presented with metastatic high-grade serous ovarian carcinoma involving the liver, peritoneum, lymph nodes, and bone. Tumor immunohistochemistry demonstrated isolated loss of MSH2 expression. Germline testing confirmed a pathogenic MSH2 mutation, establishing a diagnosis of Lynch syndrome. Following multidisciplinary discussion, combined chemoimmunotherapy was recommended; however, the patient declined cytotoxic chemotherapy and was treated with dual immune checkpoint blockade (ipilimumab and nivolumab). Early imaging demonstrated a rapid and marked metabolic response, with subsequent scans confirming near-complete resolution of disease. The patient underwent interval cytoreductive surgery, which revealed minimal residual disease. Maintenance nivolumab and bevacizumab were initiated postoperatively. At latest follow up after 2 years, the patient remains in complete radiologic remission, with no evidence of active disease.

**Conclusion:**

This case highlights the importance of comprehensive molecular profiling for patient selection and biomarker-guided treatment strategies in patients with Lynch syndrome. Despite the serous histology of the patient’s ovarian cancer, the presence of dMMR by immunohistochemistry and an underlying history of Lynch syndrome supported the use of a chemotherapy-free regimen with dual ICIs. Subsequent somatic next-generation sequencing revealed MSI-H/TMB-H disease, which explains the remarkable response that the patient had.

## Introduction

Ovarian cancer ranks as the eighth most common malignancy among females, representing approximately 3.7% of all new cancer cases and 4.7% of cancer-related deaths in 2020 (1). Standard management for advanced epithelial ovarian cancer limited to the abdomen and pelvis typically consists of cytoreductive surgery with neoadjuvant or adjuvant platinum-based chemotherapy, combined with the anti-VEGF bevacizumab (2). Despite initial chemosensitivity in many patients, durable responses are uncommon. In the ICON-7 trial, the median progression-free survival (PFS) and overall survival (OS) among patients who received carboplatin, paclitaxel, and bevacizumab were 19.4 and 58 months, respectively (3).

Microsatellite instability–high (MSI-H) or mismatch repair–deficient (dMMR) ovarian cancer represents a rare molecular subset, accounting for 1.7–2.4% of ovarian cancer diagnoses (4). MSI-H/dMMR tumors exhibit high mutational burden and increased neoantigen load. This creates an immunogenic tumor microenvironment and results in improved responses to immune checkpoint blockade (5). In MSI-H/dMMR ovarian cancer, there is limited data regarding the use of immune checkpoint inhibitors (ICIs), mostly derived from basket trials and retrospective observations. The KEYNOTE-158 trial evaluated the use of single-agent pembrolizumab in different MSI-H/dMMR malignancies. Among 15 patients with MSI-H/dMMR ovarian cancer and a median follow-up of 13.4 months, the overall response rate (ORR) was 33.3%, including 3 patients who achieved a complete response (CR) and 2 who had a partial response (PR). The median PFS was 2.2 months (95% CI: 1.9–6.2), while the median overall survival (OS) was 33.6 months (6, 7). Another trial evaluated nivolumab in 35 patients with hypermutated endometrial and ovarian malignancies, and reported an ORR of 58.8%, including CR and PR rates of 21% and 38%, respectively. Over a median follow-up of 42.1 months (range: 8.9–59.8 months), the median OS was not reached (NR). The 1-year OS rate was 79%, and the median PFS 21.6 months (8).

Lynch syndrome (LS) is an autosomal dominant hereditary cancer syndrome caused by germline pathogenic variants in mismatch repair (MMR) genes, including *MLH1*, *MSH2*, *MSH6*, *PMS2* or *EPCAM* deletions. LS is associated with a markedly increased lifetime risk of colorectal and endometrial cancers and, to a lesser extent, ovarian and other malignancies (9). Recognition of LS through family history assessment and germline testing is therefore crucial for early diagnosis, risk reduction, and personalized treatment strategies (9).

MMR/MSI status can be assessed using immunohistochemistry (IHC) for mismatch repair proteins (*MLH1, MSH2, MSH6, PMS2*), polymerase chain reaction (PCR)-based assays, or next-generation sequencing (NGS). In colorectal cancer, PCR-based MSI assessment traditionally relies on the analysis of the five National Cancer Institute (NCI)-recommended microsatellite markers, with instability in ≥2 markers indicating MSI-high (MSI-H) and instability in a single marker classified as MSI-low (MSI-L) (10). However, in non-colorectal tumors, the optimal testing strategy remains less well established. Therefore, discordant results should be interpreted using an integrated approach incorporating histopathology, IHC, germline findings, and comprehensive genomic profiling. While MSI-H/dMMR tumors have been associated with responsiveness to immune checkpoint inhibitors, the clinical behavior and treatment susceptibility of MSI-L cancers remain largely unknown and available data is limited. Generally, MSI-L tumors are considered similar to microsatellite-stable (MSS) tumors and have not been shown to respond meaningfully to immune checkpoint blockade (11). On the other hand, in patients with MSI-H/dMMR colorectal cancer, the use of ICIs was associated with improved outcomes compared with the use of chemotherapy, but this benefit was not observed in MSI-L and MSS tumors (12).

Herein, we report a case of Lynch syndrome-associated ovarian cancer which was initially reported as dMMR/MSI-L but demonstrated a marked and durable clinical response to immune checkpoint blockade without chemotherapy. Subsequent NGS testing confirmed MSI-H disease. This case highlights the importance of comprehensive molecular profiling in ovarian cancer and careful patient selection for biomarker-guided treatment regimens, particularly chemotherapy-sparing treatment strategies in selected patients.

## Case presentation

**FIGURE 1:**
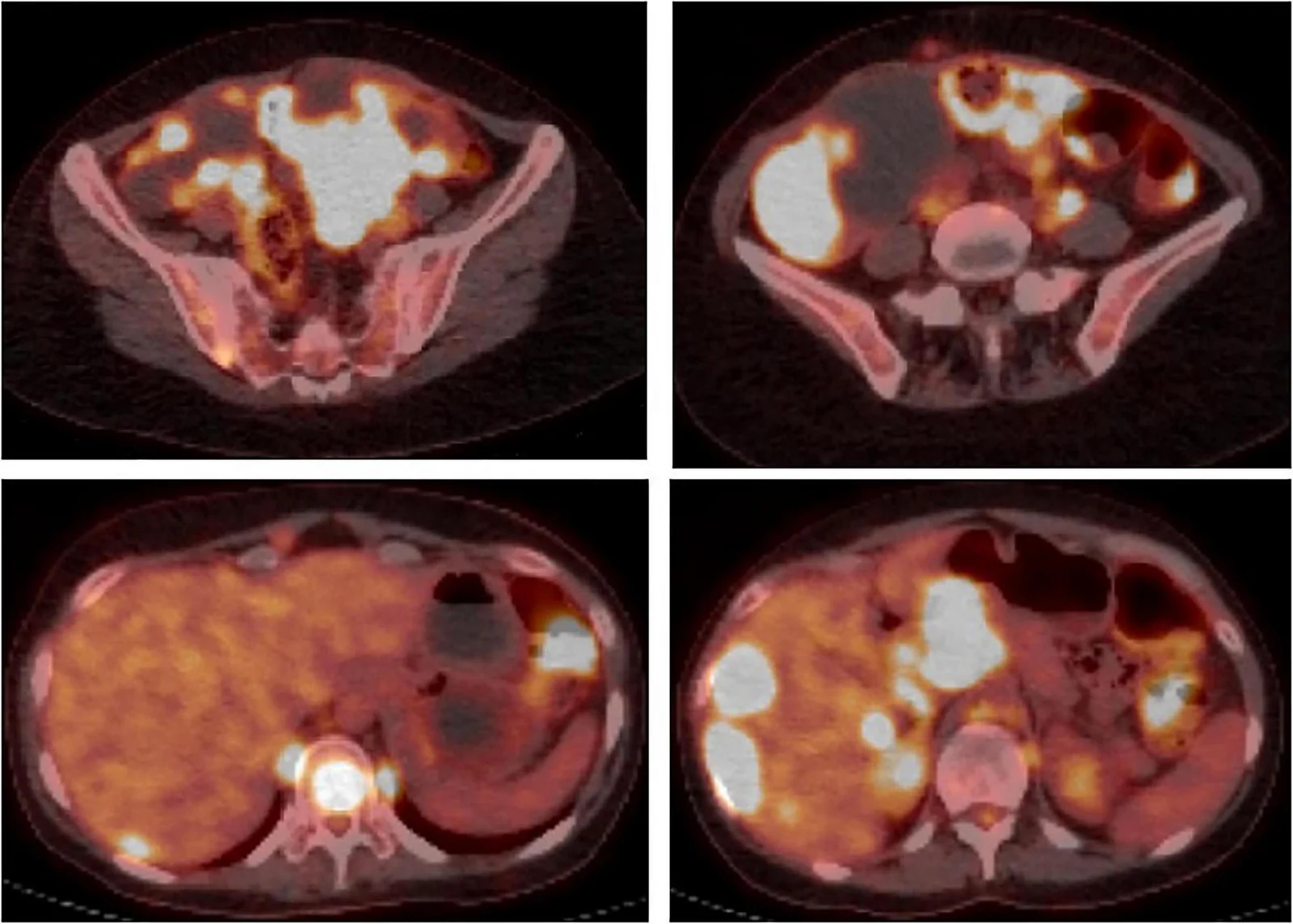
PET FDG CT scan at diagnosis.

**FIGURE 2:**
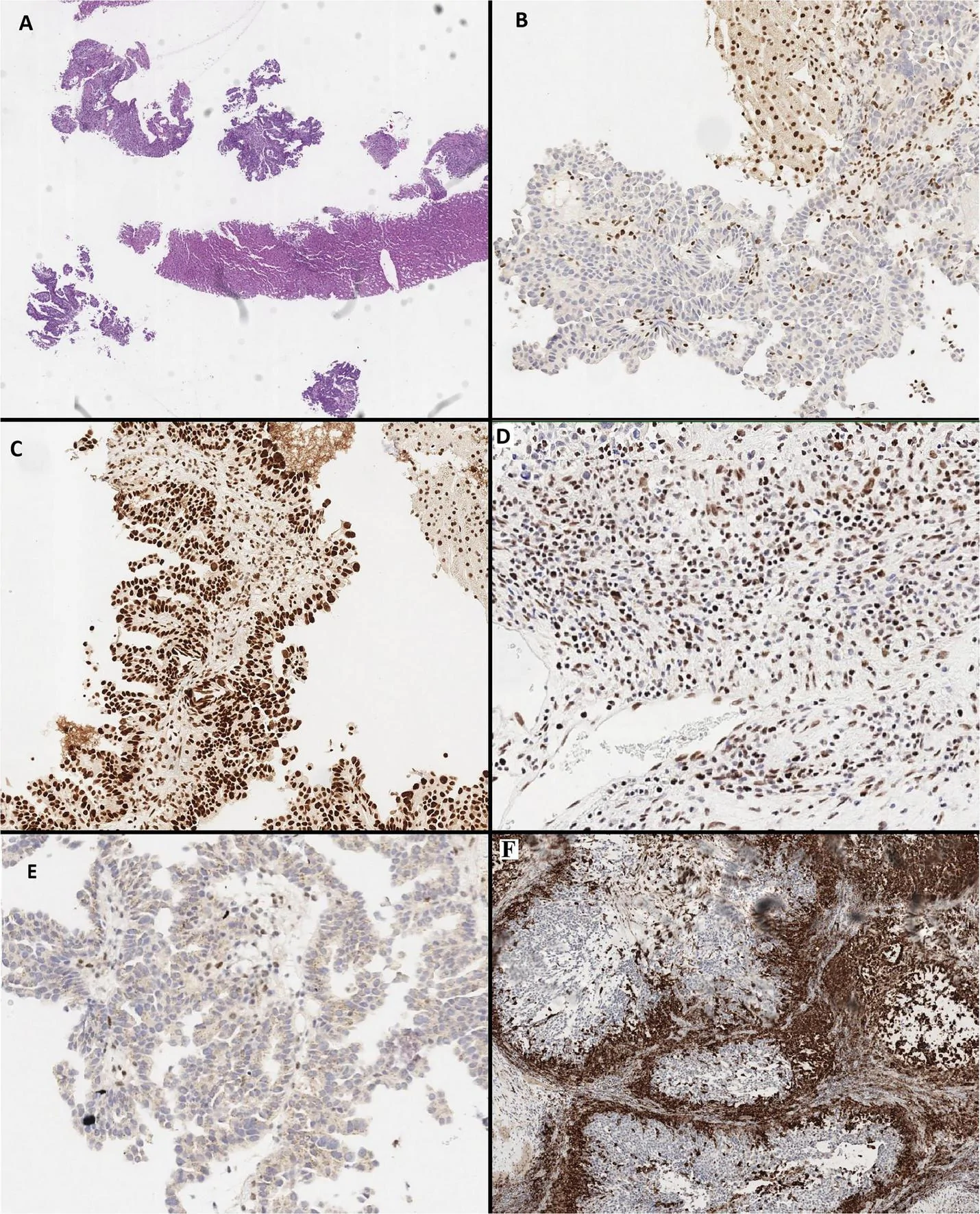
Histopathologic images of tumor tissue: **(A)** hematoxylin and eosin staining at 2.5× magnification; **(B)** loss of MSH2 expression at 12× magnification; **(C)** retained MSH6 expression at 20× magnification; **(D)** positive p16 staining; **(E)** loss of p53 expression; and **(F)** PD-L1 (22C3, Dako) staining at 20× magnification.

A 42-year-old female patient, with a negative personal medical history but a strong family history of cancer from her maternal side, including ovarian cancer in her mother, colorectal cancer in her brother, and ovarian, colorectal, and skin cancers in maternal second-degree relatives (Supplementary Figure 1), presented with bloating and self-palpated of a left lower quadrant abdominal mass. MRI of the abdomen and pelvis with intravenous contrast showed a large complex mixed enhancing solid and cystic mass, occupying almost the entire pelvic cavity along with several hepatic lesions suggestive of metastases. This was followed by an 18F-fluorodeoxyglucose positron emission tomography/computed tomography (FDG-PET/CT) scan that showed similar findings of large FDG-avid pelvic masses with areas of necrosis and multiple avid liver lesions, in addition to avid peritoneal deposits and lymph nodes, and avid bone lesions including right femoral head, right iliac bone, and T6 and T11 vertebral bodies (Figure 1). CT-guided biopsy of a liver lesion confirmed high-grade serous carcinoma of ovarian origin. IHC analysis demonstrated positive p16, negative p53 (mutated type), negative estrogen receptors, positive Wilms’ tumor 1 (WT-1), in addition to isolated loss of MSH2 expression, with intact nuclear expression of MLH1, MSH6 and PMS2 (Figure 2). The pathology report stated that the tumor is MSI-L with instability of 1 of the 5 National Cancer Institute (NCI) markers, however, a detailed PCR result that shows details of the unstable markers and the method used was not available. In view of the strong family history of cancer, the patient’s maternal cousin, who developed CRC at the age of 38, underwent germline testing using the Agilent Inherited Cancer Research Panel (United States) at Cerba laboratories (France), which showed carriership of two germline pathogenic variants: PALB2(NM_024675. 4):c.1056_1057delGA (p.Lys353IlefsTer7) and MSH2(NM_ 000251. 3):c.(1759 + 1_1760-1)_(2458 + 1_2459-1)del, p.? (deletion of exons 12 to 14). Targeted testing for the familial *PALB2* and *MSH2* variants was performed using Sanger sequencing and multiplex ligation-dependent probe amplification (MLPA) at Centogene laboratories (Germany). Our patient was found to carry the *MSH2* variant solely, consistent with a diagnosis of LS.

**FIGURE 3:**
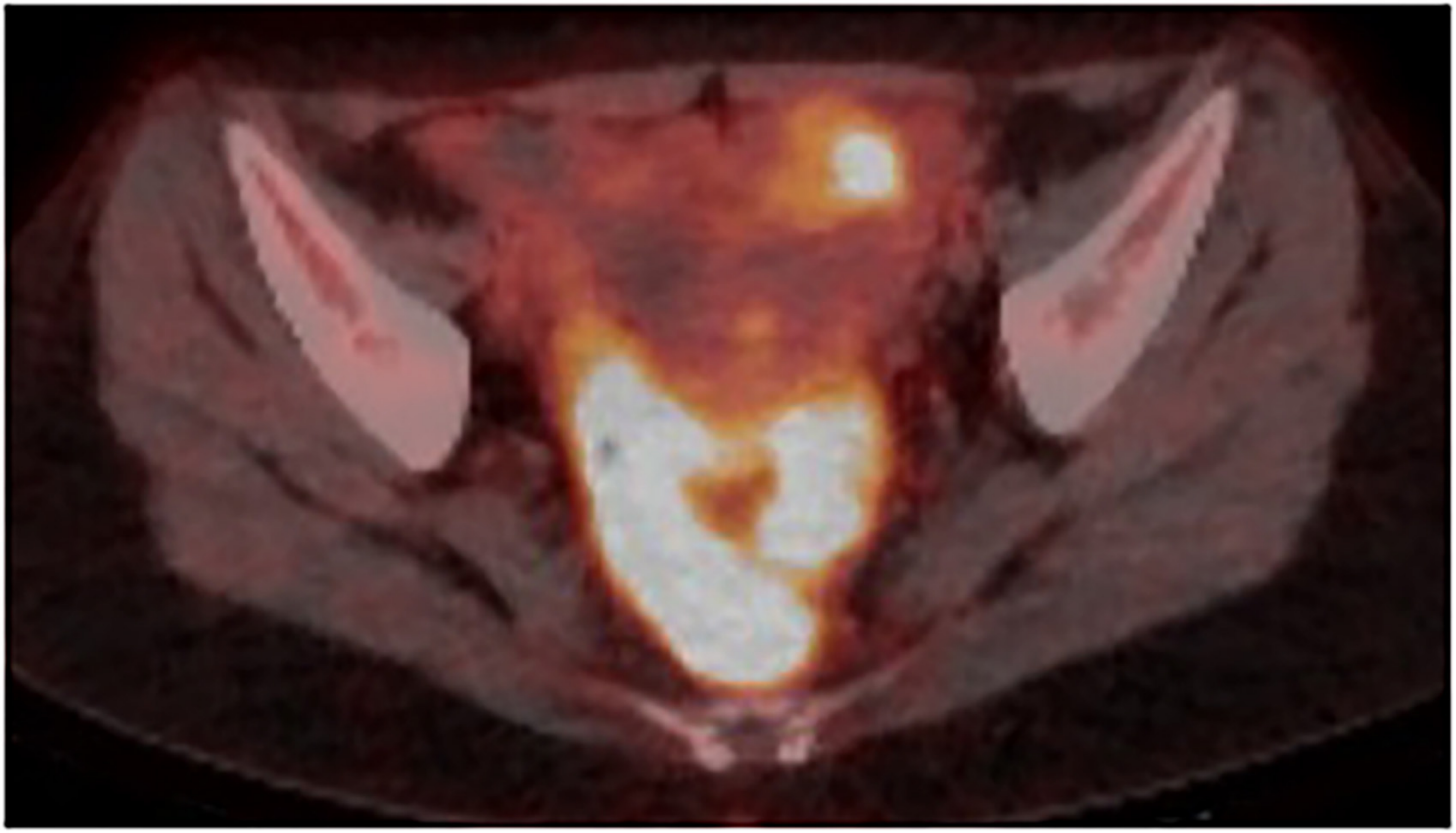
PET FDG CT scan after 4 cycles of treatment showing a new non-specific avid lesion in the left hemi-pelvis.

**FIGURE 4:**
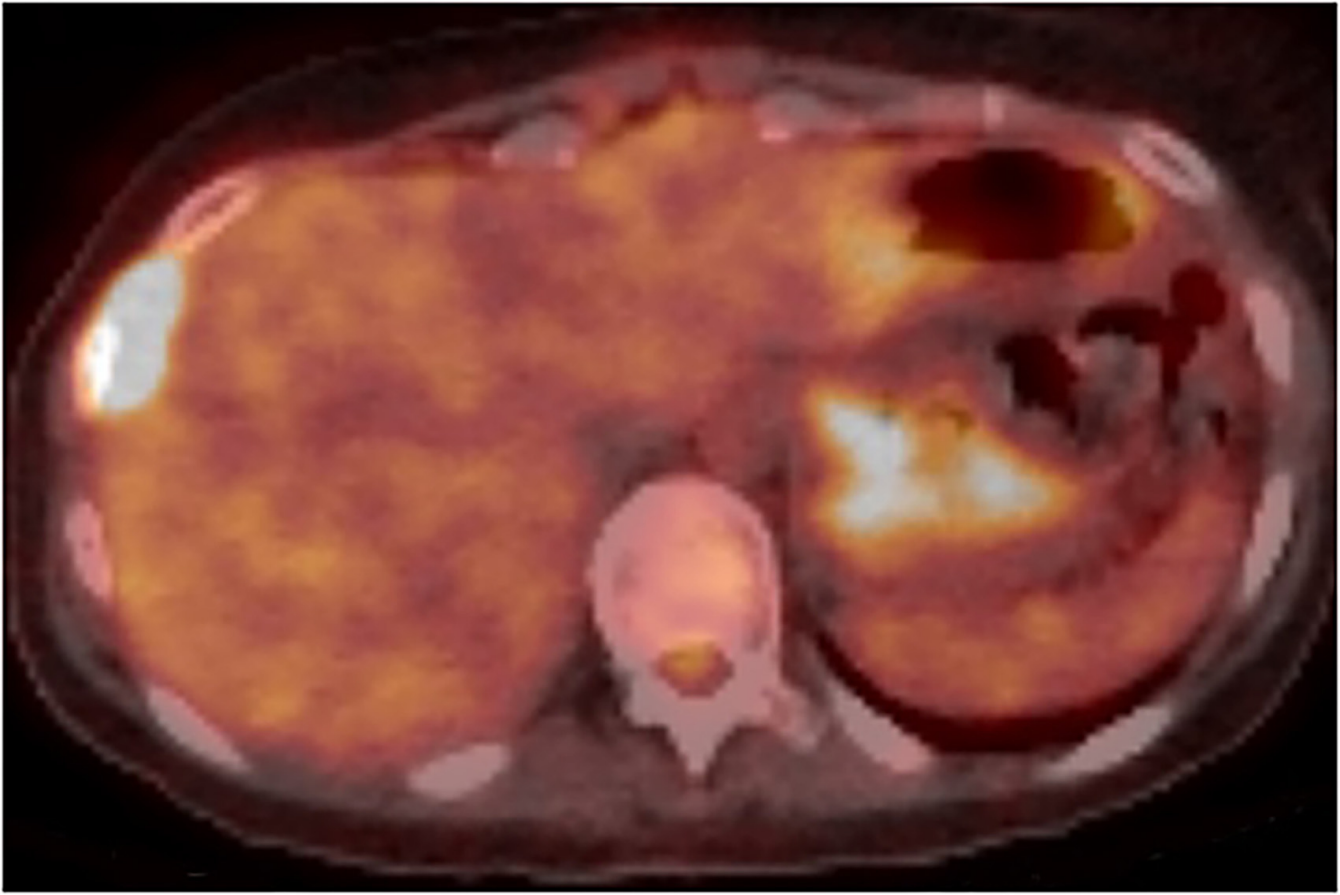
PET FDG CT scan showing an avid lesion in segment 5 of the liver.

The case was discussed at our multidisciplinary tumor board meeting, and the recommendation was to initiate treatment with chemoimmunotherapy. After discussion, the patient refused chemotherapy. Since the patient’s tumor was dMMR, the decision was made to use ICIs without chemotherapy, and thus she was started on dual immune checkpoint blockade with ipilimumab 1 mg/kg every 6 weeks and nivolumab 3 mg/kg every 2 weeks of a 42-day cycle. A follow-up FDG-PET/CT scan after 2 cycles of treatment showed significant decrease in size and resolution of activity of all lesions. The choice of dual immune checkpoint blockade was based on the patient’s dMMR phenotype by IHC, germline MSH2-associated Lynch syndrome, extensive metastatic disease, and refusal of cytotoxic chemotherapy. Although evidence in ovarian cancer remains limited, dual PD-1/CTLA-4 blockade has shown enhanced activity compared with PD-1 monotherapy in MSI-H/dMMR metastatic colorectal cancer (13). Therefore, nivolumab plus ipilimumab was selected as an individualized, biomarker-driven, chemotherapy-sparing approach after multidisciplinary discussion, with careful monitoring for immune-related toxicity. A repeat FDG-PET/CT scan after 4 cycles showed continued response to treatment with the exception of a new, non-specific focally avid lesion in the pelvis (Figure 3), with laboratory testing showing normalization of CA125 (Supplementary Figure 2). The patient underwent debulking surgery (total abdominal hysterectomy and bilateral salpingo-oophorectomy), with lymph node dissection and excision of omental, peritoneal and hepatic lesions. Pathology showed residual disease in one ovary, and the remaining tissue was negative for carcinoma (surgical stage ypT3N0M0). Nivolumab 360 mg every 3 weeks was resumed 1 week postoperatively, and bevacizumab 7.5 mg/kg was added 1 month later. The patient continues to receive both agents. Notably, 1 year later, the patient was admitted with a small bowel obstruction requiring resection of an ileal loop; pathologic examination was negative for malignancy. A few months later, the patient underwent CT-guided biopsy of an FDG-avid liver lesion seen on PET/CT (Figure 4). The pathology was negative for malignancy. The avid lesions seen on PET CT scans (Figures 3 and 4) in the absence of symptoms, decreasing tumor markers and negative pathology (both surgical and the biopsy) favored pseudoprogression, which can occur in patients receiving ICIs, rather than true disease progression. The latest FDG-PET/CT scan showed complete response. The patient continues to receive bevacizumab and nivolumab with a plan to complete 18 cycles of bevacizumab and then continue nivolumab monotherapy. Most recently, somatic NGS revealed a deletion of MSH2 exons 12–14 and MSI-H status, a high tumor mutational burden (TMB-H) with 40.98 mut/Mb, a pathogenic somatic BRCA2 alteration (p.T3085fs), and a negative homologous recombination deficiency (HRD) signature.

## Discussion

Current standard-of-care management of ovarian cancer includes cytoreductive surgery with platinum-based chemotherapy and anti-angiogenic therapy. Despite initial sensitivity to chemotherapy, the duration of response is limited, with a median PFS of 19.4 months (3). Multiple biomarkers are being investigated to improve response and survival. MSI-H/dMMR ovarian cancers represent a rare molecular subset, accounting for approximately 1.7–2.4% of cases. Evidence guiding the use of ICIs in ovarian cancer remains limited, as it is primarily derived from small, single-arm studies, although notable responses have been reported (4, 5). The use of pembrolizumab in the KEYNOTE-158 trial was shown to result in a median PFS of 2.32 months and a median OS of 33.6 months. However, the use of nivolumab in patients with hypermutated endometrial and ovarian malignancies yielded better outcomes, with a median PFS of 21.6 months and a median OS was not reached (6, 7). Table 1 summarizes published case reports of MSI-H ovarian cancers demonstrating excellent responses to ICIs in MSI-H ovarian cancer.

**TABLE 1:** Case reports of MSI-H ovarian cancers.

Author(s)	Pathology	Mutation/loss of expression	Family history	Clinical characteristics	Outcomes with ICI therapy
Lam et al. (31)	Adenocarcinoma of Mullerian origin	*MSH2* mutation	Unknown	Progressive disease after treatment with carboplatin and paclitaxel and pegylated doxorubicin, subsequently switched to paclitaxel and pembrolizumab	- Radiologic response after 9 cycles and improved performance status. - Complete response after 20 cycles
Ak et al. (32)	High-grade serous adenocarcinoma	Loss of *MLH1* and *PMS2* expression	- Father with skin cancer. - Two distant cousins with breast cancer.	Primary cytoreductive surgery and adjuvant carboplatin and paclitaxel with disease recurrence, requiring another cytoreductive surgery followed by adjuvant cisplatin, gemcitabine and bevacizumab. Subsequently treated with single-agent atezolizumab 1200 mg every 3 weeks.	- CA-125 normalized after 3 cycles. - Radiologic near-complete response after 4 cycles. - Disease-free after 7 months of treatment.
Chinczewski et al. (33)	High-grade serous adenocarcinoma	*MSH2* gene mutation. Loss of *MSH2* and *MSH6* expression	Mother with colorectal cancer	Primary cytoreductive surgery with disease recurrence 5 weeks postoperatively prior to any adjuvant chemotherapy. Started on weekly carboplatin and paclitaxel with initial response but progression after 10 weeks of treatment. Lastly started on pembrolizumab 200 mg every 3 weeks.	- A radiologic partial response seen after 4 cycles and later had complete response. - Remained disease-free after 28 cycles
Yu et al. (34)	- High-grade adenocarcinoma of Mullerian origin. - Well-differentiated rectal tubular adenocarcinoma.	*MSH2* mutation	- Father with intestinal cancer. - Second uncle with hepatocellular carcinoma. - Second aunt with cervical and gastric cancer. - Second, third, fifth, and seventh cousins with intestinal cancer.	Diagnosis of advanced synchronous ovarian and rectal cancers. Started treatment on carboplatin, paclitaxel and sindilizumab 200 mg every 3 weeks for 4 cycles, followed by surgery then another 4 cycles then ICI maintenance.	Partial radiologic response after treatment, then subsequent surgery showed minimal residual rectal tumor (pTis, N0) and a 0.1 cm minimal residual ovarian tumor. - R0 resection was achieved. - Remained disease-free after 1 year and 8 months of treatment.
Daniyal et al. (35)	Carcinosarcoma	*MSH6* mutation Loss of *MSH6* expression	- Mother with ovarian cancer. - Father with prostate cancer.	Initially had a suboptimal debulking surgery followed by carboplatin and paclitaxel, and had with disease progression after 6 cycles. She had further disease progression 2 months after starting single-agent bevacizumab. She was then started on pembrolizumab 200 mg every 3 weeks with stable disease for 3 years then progression, for which cyclophosphamide was added	Stable disease for more than 3 years of pembrolizumab.

In our case, the identification of dMMR enabled selection of a chemotherapy-sparing, biomarker-driven treatment approach, using dual ICIs with the anti-CTLA-4 ipilimumab and the anti-PD-1 nivolumab, given the patient’s extensive metastatic involvement at presentation, including liver, bone, peritoneal, and pelvic lesions, and the patient’s preference to avoid the morbidity of cytotoxic therapy. The decision to use dual immunotherapy with ipilimumab and nivolumab, followed by nivolumab maintenance was based on the success of this regimen in dMMR/MSI-H metastatic colorectal cancer in the CheckMate 142 trial, which reported an objective response rate of 69% and disease-control rate of 84% (14). Our patient tolerated dual immunotherapy well, without any immune-related adverse events, and had exceptional clinical and pathologic responses, prompting continuation of single-agent nivolumab as maintenance therapy postoperatively. Bevacizumab was added to maintenance therapy despite the absence of direct evidence supporting its use following neoadjuvant dual immune checkpoint blockade. Bevacizumab has primarily been evaluated in combination with platinum-based chemotherapy in the neoadjuvant or adjuvant setting, followed by maintenance. Its clinical benefit, acceptable safety profile, and potential immunomodulatory effects through vascular normalization provided the rationale for its inclusion in our patient’s individualized treatment strategy (15, 16).

Serous histology is not only rare in LS but also clinically notable because high-grade serous ovarian carcinoma has generally shown limited responsiveness to single-agent immune checkpoint inhibition in ovarian cancer populations. This is due to the fact that these tumors are mostly MSS and have low TMB (17). Trials that enrolled unselected patients with ovarian adenocarcinoma have reported disappointing response rates. For instance, in the KEYNOTE-100 trial and the ovarian cohort of JAVELIN Solid Tumor trial, the use of single-agent pembrolizumab and avelumab led to response rates of less than 10%, with the highest response rates observed in clear cell carcinoma (18, 19). However, in the presence of molecular alterations such as BRCA mutations, POLE mutations, and MSI-H/dMMR, these tumors exhibit increased immune cell infiltration, increased neoantigen load and enhanced expression of immune checkpoint molecules within their microenvironment. These immunogenic features may underlie the improved response to ICIs observed in these patients. Consequently, these molecular alterations have emerged as important predictive biomarkers for response to ICI therapy (20, 21). Our patient was diagnosed with high-grade serous ovarian adenocarcinoma, however, subsequent testing confirmed MSI-H/dMMR, TMB-H and BRCA2 mutation; all of which are associated with the remarkable response to immune checkpoint blockade which our patient had.

MSI-H and/or TMB-high status usually results from a null somatic or germline pathogenic variant in an MMR gene or methylation of the *MLH1* promoter (22, 23). According to the two-hit hypothesis, tumor development in LS requires a second somatic event, most commonly loss of heterozygosity or a somatic mutation in the same MMR gene. Historically, MSI-H/dMMR is considered a predictive biomarker of ICI response, while MSI-L is generally not associated with immunotherapy sensitivity (12). Tumor sequencing studies have demonstrated that a somatic second hit can be identified in the majority of LS-associated tumors, including up to 70% of *MSH2* carriers (24). The nature of this second hit may not generate the typical insertion–deletion signatures. In the setting of suspected LS, NGS plays a critical role in identifying somatic second-hit alterations in mismatch repair genes (24).

The patient’s tumor demonstrated isolated loss of MSH2 protein on IHC (deficient MMR). Typically, loss of MSH2 expression is known to be associated with concurrent loss of MSH6, while the loss of MLH1 accompanies the loss of PMS2. Consequently, some centers use a cost-saving two-antibody screening approach based only on IHC staining for MSH6 and PMS2. Although isolated loss of MSH2 is unusual, our case demonstrates the need for screening using all four proteins to avoid missing therapeutic opportunities or germline alterations associated with LS. In concordance, Pearlman et al. identified 11 of 33 tumors (33%) with MSH2 loss and intact MSH6 expression (25). Furthermore, the complementarity of IHC and molecular-based testing is essential for optimal LS screening, especially for *MSH2* carriers in whom IHC and PCR testing were found to achieve sensitivities of 94% and 93%, respectively (26). As such, single-gene *MSH2* sequencing is recommended in the event of MSH2 expression loss, regardless of MSI testing results (9).

Another intriguing aspect of our case is the serous histology of the tumor. High-grade serous carcinoma represents an uncommon histologic subtype within the spectrum of Lynch syndrome (LS)-associated ovarian carcinomas. LS-associated ovarian cancers typically have endometrioid, mixed endometrioid, or clear cell histology. In contrast, high-grade serous carcinoma is linked to BRCA mutations rather than LS. Nevertheless, rare high-grade serous adenocarcinomas have been reported in confirmed LS carriers, including a series described by Ryan et al. (27, 28). In these patients, MMR and MSI testing may be discordant due to several biologic and technical factors. Conventional MSI-PCR testing was developed primarily in colorectal cancer, initially using the NCI/Bethesda marker panel and later mononucleotide panels, with MSI-H thresholds largely derived from colorectal cohorts (10). However, MSI is not a uniform molecular phenotype across tissues. Tumors arising in different organs may differ in the microsatellite loci affected, the proportion of unstable loci, and the magnitude of allelic shifts, which may affect the sensitivity of fixed PCR panels. This issue is particularly relevant in ovarian carcinoma, where MSI-H/dMMR is uncommon, enriched mainly in endometrioid and clear cell histologies, and is rare in high-grade serous carcinoma. Studies evaluating ovarian tumors have shown that discordance between IHC, PCR, NGS, and MMR gene mutation status can occur, including MMR-deficient tumors that are not classified as MSI-H by PCR (29). In ovarian cancer, MSH2-deficient ovarian tumors may generate instability patterns that are atypical for colorectal cancer, with instability affecting loci not included in standard PCR panels or producing allelic shifts that are insufficient to meet assay-specific MSI-H thresholds (30). Therefore, in ovarian tumors with strong clinicopathologic or hereditary evidence of dMMR, PCR-based MSI results should not be interpreted in isolation. Rather, an integrated assessment with four-protein MMR IHC, germline testing, and somatic NGS may provide a more accurate basis for identifying patients likely to benefit from immune checkpoint blockade.

This case underscores the importance of careful assessment of family history and timely genetic testing in patients with suspected LS, particularly in younger individuals. The diagnosis of ovarian cancer in our patient in her early 40s, given the presence of two germline variants in the family, highlights the need for heightened clinical suspicion and early referral for genetic counseling (9). The lifetime risk of LS-associated cancers varies according to the affected mismatch repair protein, although colorectal and endometrial cancers remain the most prevalent across all genotypes. The risk of ovarian cancer is highest in *MSH2* and *MLH1*-associated LS, reaching up to 38% in *MSH2* carriers, with a median age at diagnosis of 43 years (9). Given the elevated risk of endometrial cancer in those individuals, risk-reducing total abdominal hysterectomy and bilateral salpingo-oophorectomy (TAHBSO) should be considered starting at the age of 40 years (9). Genetic counseling is essential for reducing cancer-related risks, particularly for LS-associated cancers beyond the gastrointestinal and gynecologic systems, such as urothelial, pancreatic, prostate, breast, brain, skin cancers and sarcomas. These risks depend on the specific pathogenic variant and the patient’s personal and family histories. As a result, surveillance and risk-reducing strategies are individualized and tailored after assessment by a genetic counselor (9).

## Conclusion

This case describes a Lynch syndrome–associated serous ovarian carcinoma with a remarkable response to ICIs. The durable response to dual immune checkpoint blockade without chemotherapy supports the importance of integrated biomarker assessment, including IHC, germline testing, and somatic NGS.

## Data Availability

The raw data supporting the conclusions of this article will be made available by the authors, upon reasonable request.

## Supplementary material

Supplementary Figure 1 Pedigree.

Supplementary Figure 2 CA-125 trend during treatment.
